# Effect of age and autism spectrum disorder on oxytocin receptor density in the human basal forebrain and midbrain

**DOI:** 10.1038/s41398-018-0315-3

**Published:** 2018-12-04

**Authors:** Sara M. Freeman, Michelle C. Palumbo, Rebecca H. Lawrence, Aaron L. Smith, Mark M. Goodman, Karen L. Bales

**Affiliations:** 1 Department of Psychology, California National Primate Research Center, University of California-Davis, One Shields Avenue, Davis, CA 95616 USA; 2 Silvio O. Conte Center for Oxytocin and Social Cognition, Center for Translational Social Neuroscience, Department of Psychiatry and Behavioral Sciences, Yerkes National Primate Research Center, Emory University, 954 Gatewood Road, Atlanta, GA 30329 USA; 3 Department of Radiology and Imaging Sciences, Emory University, 1841 Clifton Road, Atlanta, GA 30322 USA

## Abstract

The prosocial hormone oxytocin (OXT) has become a new target for research on the etiology and treatment of autism spectrum disorder (ASD), a condition characterized by deficits in social function. However, it remains unknown whether there are alterations in OXT receptor (OXTR) levels in the ASD brain. This study quantified the density of OXTR and of the structurally related vasopressin 1a receptor (AVPR1a) in postmortem brain tissue from individuals with ASD and typically developing individuals. We analyzed two regions known to contain OXTR across all primates studied to date: the nucleus basalis of Meynert (NBM), which mediates visual attention, and the superior colliculus, which controls gaze direction. In the NBM specimens, we also analyzed the neighboring ventral pallidum (VP) and the external segment of the globus pallidus. In the superior colliculus specimens, we also analyzed the adjacent periaqueductal gray. We detected dense OXTR binding in the human NBM and VP and moderate to low OXTR binding in the human globus pallidus, superior colliculus, and periaqueductal gray. AVPR1a binding was negligible across all five regions in all specimens. Compared to controls, ASD specimens exhibited significantly higher OXTR binding in the NBM and significantly lower OXTR binding in the VP, an area in the mesolimbic reward pathway. There was no effect of ASD on OXTR binding in the globus pallidus, superior colliculus, or periaqueductal gray. We also found a significant negative correlation between age and OXTR binding in the VP across all specimens. Further analysis revealed a peak in OXTR binding in the VP in early childhood of typically developing individuals, which was absent in ASD. This pattern suggests a possible early life critical period, which is lacking in ASD, where this important reward area becomes maximally sensitive to OXT binding. These results provide unique neurobiological insight into human social development and the social symptoms of ASD.

## Introduction

Extensive research has established the potent ability of the hypothalamic neuropeptide oxytocin (OXT) to modulate social behavior across species, including humans^1^. Convincing results from animal research led to examinations of the effects of OXT administration to humans, as well as a variety of investigations into the possible involvement of the OXT system in the etiology and treatment of psychiatric disorders, especially autism spectrum disorder (ASD)^2^. ASD is a heterogeneous condition characterized by restricted interests/behaviors and deficits in social communication, and it affects approximately 1 in 59 children in the United States^3^. Because social symptoms are at the core of this disorder, the OXT system has been implicated in the biology of ASD and has become a promising treatment option for the social symptoms of ASD. The increased research effort into the potential involvement of OXT and its receptor (OXTR) in ASD includes genetic studies, analyses of OXT levels in biological fluids, and assessments of OXT treatment, which will be described further below. Although neurobiological studies on the OXT system in ASD, such as the current study, are more rare than these system-wide investigations, they contribute essential information to the larger body of work by providing direct assessments of the ASD brain.

To initially address the possible involvement of OXT in the biological underpinnings of ASD, several studies examined OXT-related genes in clinical and non-clinical populations. Indeed, many of these genetic studies have found that polymorphisms in the human OXT receptor (*OXTR*) gene are significantly associated with an ASD diagnosis and/or predict the severity of ASD^4–12^. Results from studies in non-ASD populations have also linked *OXTR* polymorphisms to other measures of social behavior, such as empathy^13^, prosocial temperament^14^, and pair-bonding behavior^15^. Although some of these reports are conflicting^16^, the results of these genetic studies provide important, yet indirect, support for the potential involvement of the OXT system in complex social cognition in humans, including populations with ASD.

These genetic investigations have been complemented by hormone studies that have examined whether circulating levels of plasma OXT differ between patients with ASD and matched controls and whether these baseline plasma OXT levels correlate with symptom severity. While several studies have found lower baseline levels of OXT in plasma in children with ASD compared to controls^17–19^, recent studies with larger sample sizes have found no significant difference in plasma OXT between individuals with ASD and controls^5,20^. However, one of these papers found significant positive associations between plasma OXT and sociality in patients with ASD, unaffected siblings, and unrelated neurotypical controls alike. Specifically, plasma OXT levels positively predicted performance on a theory of mind task as well as social communication scores across all groups^5^. This study also found that plasma OXT levels were highly heritable across the ASD/unaffected sibling dyads, further linking the involvement of the OXT system with the underlying biology of sociality in humans. It has also been reported that plasma OXT levels were positively correlated with ASD symptom severity among ASD samples, but did not differ between ASD and control groups^21^. Given these mixed findings, it is important to note that there are two active controversies regarding the measurement of OXT in biological fluids. First, there is as yet no consensus on the best methodology to measure OXT^22,23^, and second, it is unclear whether these peripheral measures of OXT are accurate approximations for levels of OXT in the central nervous system^24^. Although a recent meta-analysis across 516 participants reported no significant association between baseline levels of OXT in plasma and cerebrospinal fluid^25^, this study did not include participants with ASD, so further research is warranted.

In order to extend these peripheral studies of genetics and hormone levels into research that focuses on the brain and behavior, there has been increasing use of intranasally administered OXT (IN-OXT) as an experimental therapeutic to target and ameliorate the social symptoms in patients with ASD. Clinical studies designed to test the effectiveness of IN-OXT treatment in adults and children with ASD (including high-functioning and low-functioning populations) have shown mixed results. Several studies report that IN-OXT can improve some aspects of social functioning in ASD, including enhancing the saliency of human faces^26^, improving emotion recognition^27^, and increasing feelings of social reciprocity and increasing gaze to the eyes^19^. However, these studies involved acute dosing, rather than the long-term, daily treatment that would realistically be used in a therapeutic setting. Studies employing longer, daily dosing paradigms for IN-OXT delivery to individuals with ASD have found IN-OXT to be safe, but generally without substantial behavioral effects^28–31^. Despite these variations in reported findings, clinical trials of IN-OXT for use in ASD are in progress, and some subtle yet positive effects of chronic IN-OXT treatment on social responsiveness in ASD have been reported^32,33^.

Taking these biological studies of the OXT system in ASD together, it is important to remember that they all have been conducted without knowledge of the neural targets of OXT in the human brain or the mechanism by which OXT enhances human social function. Specifically, it is currently unknown whether differences in OXTR distributions in the brain exist between patients with ASD and non-clinical populations. The aim of the current study was to employ a pharmacologically optimized method to reliably visualize and measure OXTR densities in postmortem human brain tissue^34^ from patients with ASD and from typically developing (TD) controls.

The neuroanatomical map of OXTR binding in the human brain has not been reliably characterized, except for two outdated studies^35,36^ whose results have recently been called into question^34^ due to the radioligand’s high degree of cross-reactivity to the structurally similar vasopressin 1a receptor (AVPR1a). To confirm the specific detection of OXTR, we measured both OXTR and AVPR1a with an optimized and reliable method previously validated in nonhuman primate and human brain tissue^34,37,38^. We took a comparative approach to identify regions of interest (ROI) for our study and used existing information from the OXTR distributions of the brains of humans and nonhuman primates. Because OXTR binding in primate brains is limited to a small number of subcortical areas and putatively absent in the cortex^35,39^, we did not include cortical areas in the present study. Instead, we targeted the two areas that have been shown to contain OXTR across all nonhuman primates studied to date^39^: the nucleus basalis of Meynert (NBM) in the basal forebrain and the superior colliculus (SC) of the midbrain. These regions also have functions that are highly relevant to human social behavior and ASD symptomology. The NBM mediates selective and sustained visual attention^40,41^, and the SC has an important role in controlling eye movements and shifting gaze direction^42–44^. Patients with ASD have been shown to have altered gaze patterns and reduced attention to social imagery^45–47^. We also included in our analysis all regions that were adjacent to these two ROI and consistently identifiable across specimens, specifically the ventral pallidum (VP) and globus pallidus (GP) in the basal forebrain specimens, and the periaqueductal gray (PAG) in the midbrain specimens. To our knowledge, this study is the first to determine whether OXTR binding densities are altered in the ASD brain, with a focus on multiple behaviorally and clinically relevant subcortical regions of the human brain.

## Materials and methods

### Specimens

Unfixed, frozen blocks of de-identified human brain tissue (*n* = 44) were provided by the University of Maryland Brain and Tissue Bank, which is a Brain and Tissue Repository of the NIH NeuroBioBank. The details of this study were reviewed by the Institutional Review Board (IRB) at the University of California Davis and were found not to meet the criteria for human subjects research. The Institutional Biosafety Committee at the University of California Davis has approved this research under the Biological Use Authorization #R1840.

The TD specimens (*n* = 22) and ASD specimens (*n* = 22) were selected with recommendations and approval from the staff of the NeuroBioBank to match as best as possible on age, race, and sex. Comparisons of the demographic information and causes of death between the TD and ASP specimens are provided in Supplementary Tables 1–4. In addition to matching the ages of the ASD specimens, the ages of the TD specimens were chosen to address the effect of age on receptor binding levels. To this end, the TD specimens were selected to provide two to three samples per age group for six, sequential, 4-year age groups from 2 to 25 years of age and a seventh group of <1 year.

### Tissue preparation and anatomical details

Details about the tissue processing protocol is provided online by the NIH NeuroBioBank. Briefly, brains were chilled in wet ice prior to sectioning. The cerebrum was sectioned into left and right hemispheres. The left hemisphere was sectioned coronally, at approximately 1 cm intervals. As each section was made, it was rinsed with water, blotted dry, assigned a sequential numeric identifier, and placed in a freezing bath. All tissue blocks were frozen in isopentane/dry ice or liquid nitrogen, placed in sealed, labeled bags, and stored at −80 °C. Although there was some variability between the tissue blocks in the precise locations of each cut that was made by the neuropathologists when the tissues were originally processed and banked, the blocks encompassed the same targeted areas of the human brain: either the ventral forebrain or the midbrain.

The ventral forebrain blocks included the NBM as well as some adjacent areas of interest, such as the VP and the external segment of the GP, which were consistently identifiable across specimens and therefore included in our analysis opportunistically due to their proximity to our target. The ventral forebrain blocks did include some additional brain regions, such as midline hypothalamic nuclei, that were either incomplete or otherwise indiscernible due to edge effects and the lack of larger anatomical landmarks, without which we could not confidently confirm the regions’ identities for inclusion in the present study. Similarly, the midbrain specimen blocks that included the SC also contained the neighboring PAG, which we included in our analysis opportunistically. A small number of midbrain specimens also included incomplete portions of the red nucleus and/or substantia nigra, but those specimens were too few for those additional regions to be included in the current study.

Upon receipt from the brain bank, the unfixed, frozen blocks of brain tissue were stored at −80 °C until cryosectioning. Brain blocks were brought up to −20 °C, then sectioned at 20μm on a cryostat, and finally mounted on Fisher Frost-plus slides. The tissue blocks varied in thickness between approximately 0.5–1 cm and were sectioned into 12 adjacent series. Each block generated between 120 and 240 sections, or approximately 10 and 20 sections per series. Slides were stored in a sealed slide box with a desiccant and kept at −80 °C until use for receptor autoradiography.

### Competitive-binding receptor autoradiography

Until recent years, it was unreliable to map the locations of OXTR and the closely related AVPR1a in primate brain tissue by using the commercially available iodinated radioligands alone—^125^I-ornithine vasotocin analog for OXTR (^125^I-OVTA) and ^125^I-linearized vasopressin antagonist for AVPR1a (^125^I-LVA). This is due to the structural homology between OXTR and AVPR1a and the resulting pharmacological cross-reactivity in this system^48^. These radioligands are now known to bind to both receptor types when they are used in primate brain^37^. To remedy this issue, we developed the first reliable method for the visualization of OXTR and AVPR1a in primate brain tissue, by using a pharmacologically optimized modification to receptor autoradiography in which we co-incubate the tissue with the radioligand and a selective, unlabeled competitor compound. This approach has now been validated in postmortem brain tissue from monkeys^37,38^ and humans^34^ to selectively reveal OXTR or AVPR1a.

In the current study, we used this validated and pharmacologically optimized method of competitive-binding receptor autoradiography to identify the specific locations of OXTR and AVPR1a binding in the human brain. The specifics of this method have previously been published^34^ and are summarized briefly here. After being thawed, fixed lightly in 0.1% paraformaldehyde, and rinsed with Tris buffer, sections were incubated with either 90 pM ^125^I-OVTA or 30 pM ^125^I-LVA (PerkinElmer, Waltham, MA, USA), with or without a selective OXTR or AVPR1a competitor. Specifically, four adjacent sets of sections were incubated in the following four conditions: (1) ^125^I-OVTA alone, or (2) ^125^I-OVTA plus 1 μM of the human-selective OXTR antagonist ALS-II-69^49^, (3) ^125^I-LVA alone, (4) ^125^I-LVA plus 10 nM of the AVPR1a-selective antagonist SR49059^50^. ALS-II-69 was synthesized and provided by Dr. Aaron L. Smith. SR49059 is available from Tocris (Minneapolis, MN, USA). The slides were washed again with Tris buffer, air dried, and exposed to Carestream BioMax MR film (Kodak, Rochester, NY, USA) for 10 days, and then developed and analyzed.

### AChE counterstain and neuroanatomical analysis

**Fig. 1:**
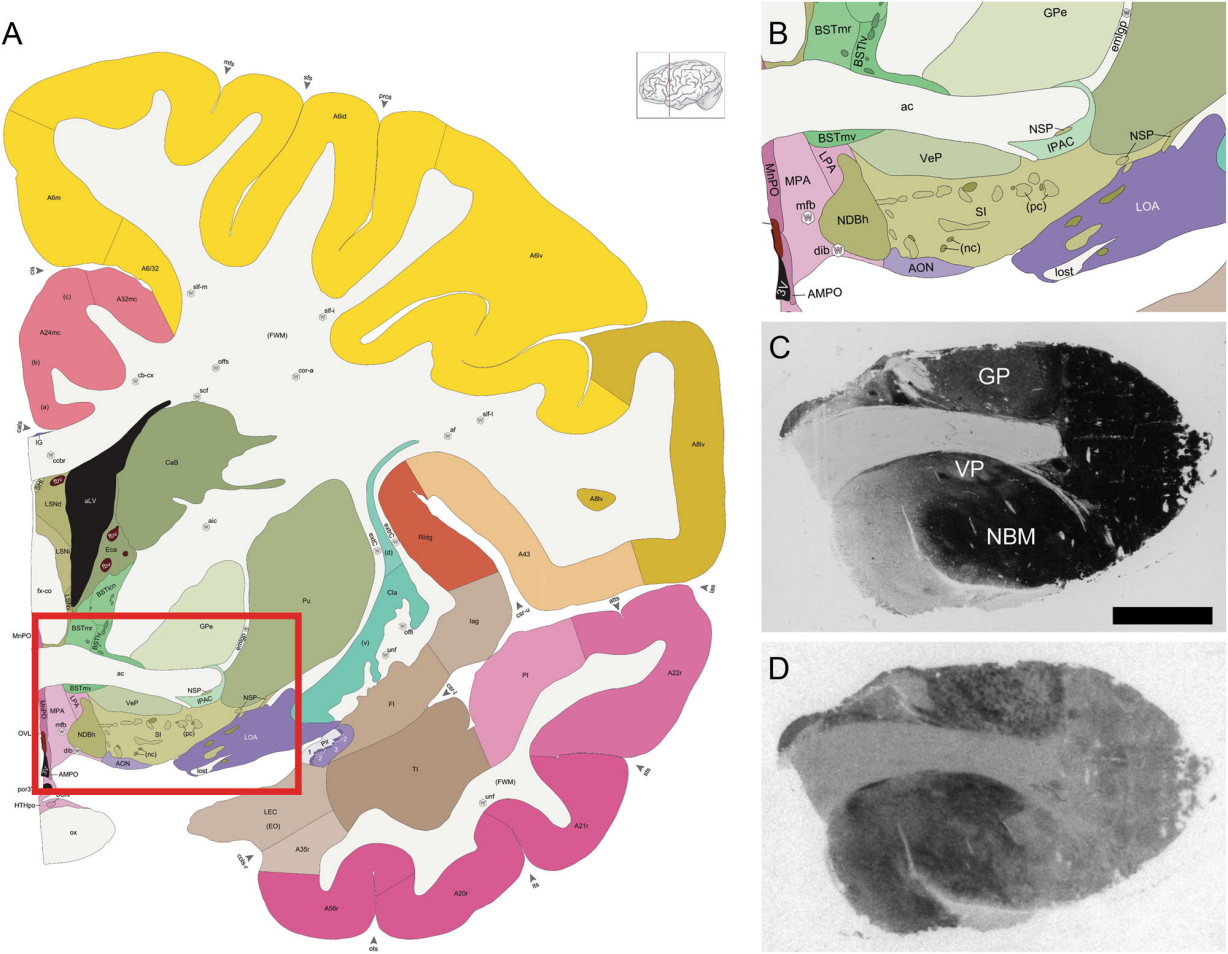
Regions of interest in the human basal forebrain. **a** An atlas plate in the coronal plane showing the relative position of the forebrain specimens (red box). **b** Zoomed in portion of the red box in **a**, showing the specific locations of the nucleus basalis of Meynert (NBM), ventral pallidum (VP), and globus pallidus (GP). Atlas images (**a**, **b**) are available under the Creative Commons License Attribution-NonCommercial-NoDerivs License and were originally published in ref. ^52^. **c** Acetylcholinesterase staining in a representative basal forebrain specimen. **d** Binding of the OXTR radioligand alone in an adjacent section from the same specimen shown in **c**. Scale bar for **c** and **d** = 25 μm

**Fig. 2:**
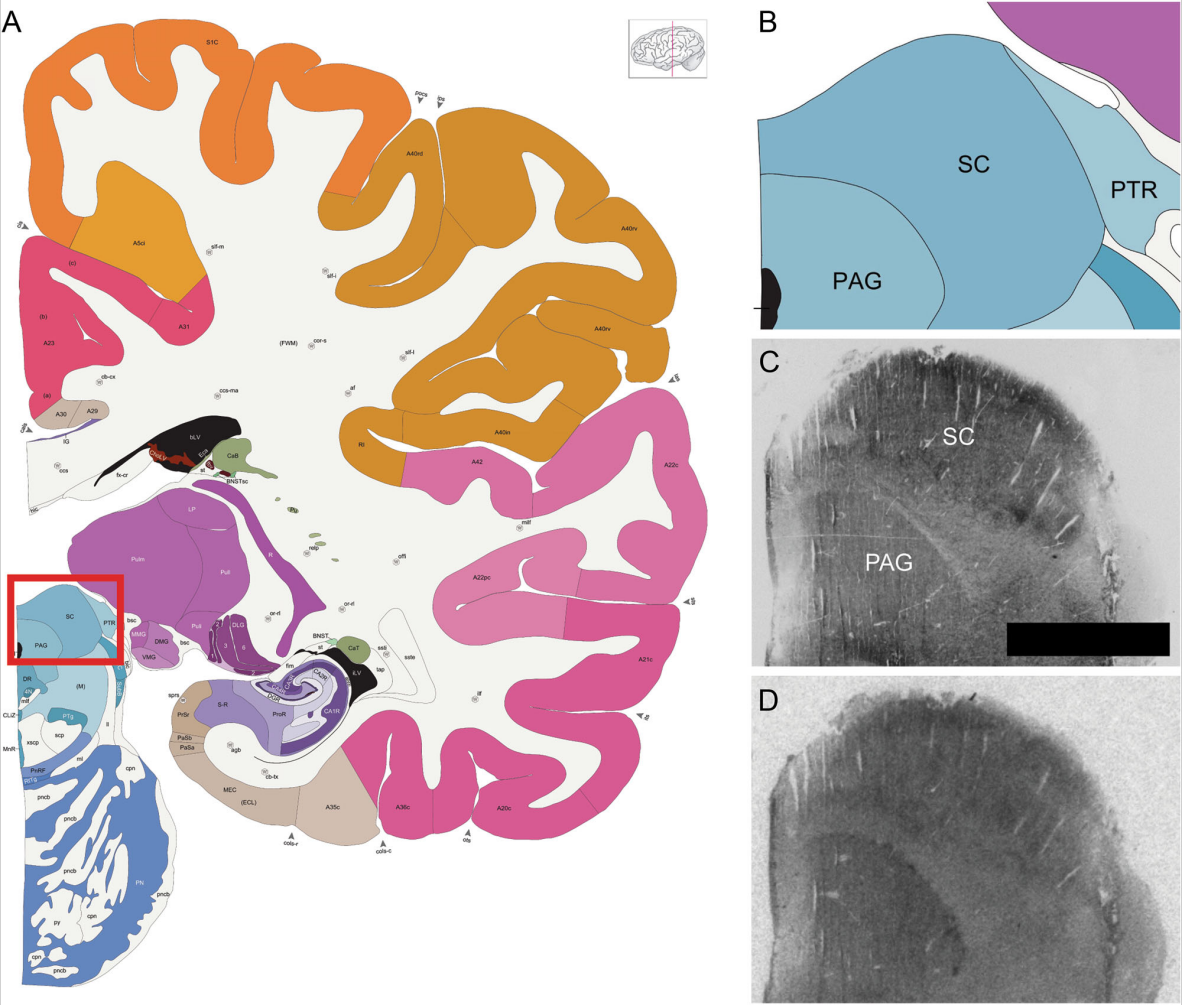
Regions of interest in the human midbrain. **a** An atlas plate in the coronal plane showing the relative position of the midbrain specimens (red box). **b** Zoomed in portion of the red box in **a**, showing the specific locations of the superior colliculus (SC) and periaqueductal gray (PAG). Atlas images (**a**, **b**) are available under the Creative Commons License Attribution-NonCommercial-NoDerivs License and were originally published in ref. ^52^. **c** Acetylcholinesterase staining in a representative midbrain specimen. **d** Binding of the OXTR radioligand alone in an adjacent section from the same specimen shown in **c**. Scale bar for **c** and **d** = 25 μm

After film development, slides were stained for acetylcholinesterase (AChE), a common neuroanatomical counterstain to aid in the specific identification of anatomical regions. Slides were processed as described previously^34,51^ and compared to the most detailed and up-to-date atlas of the human brain^52^ to confirm the anatomical boundaries of our five ROI: the NBM, VP, and GP in the basal forebrain (Fig. 1), and the SC and PAG in the midbrain (Fig. 2). GP in our study refers to the external segment of the GP.

### Quantification

Experimenters were blind to specimen diagnosis during quantification. The optical binding density (OBD) was quantified using MCID Core Digital Densitometry system (Cambridge, UK). OBD values from a set of ten ^125^I autoradiography standards (American Radiolabeled Chemicals Inc., St. Louis, MO, USA) were loaded into the software and used to generate a standard curve, from which OBD values for each ROI were extrapolated. For each specimen, OBD values were calculated for each ROI, as well as for one background area where no binding was detected. Three separate measurements for each ROI were averaged. Three measurements of background binding were also averaged and subtracted from the ROI measurements to yield normalized OBDs across specimens and correct for individual variation in background binding across specimens.

### Statistical analysis

In each ROI, we used a two-way analysis if variance with the Holm–Šídák post hoc test to determine which ROIs contained significantly higher binding of the OXTR radioligand compared to the AVPR1a radioligand and to the competitor conditions; the *p* values were also adjusted for multiple comparisons. Because we found no appreciable binding of the AVPR1a radioligand in any ROI, the remainder of our analyses were performed on a dataset that containing only the values for OXTR radioligand alone in every brain region. A Generalized Liner Model was used with this dataset to determine the effect of the following factors and interactions on normalized OBD in each ROI: diagnosis (TD and ASD), sex (male and female), race (African-American, Caucasian, and Hispanic), age, brain pH, length of time in storage, postmortem interval (PMI), sex×diagnosis interaction, and age×diagnosis interaction. Backward selection was used to remove factors from the model that were found not to be significant. Specimen ID was included as a random factor to account for individual differences. Residuals were tested for normality. If residuals were not normally distributed, a square root transformation was applied. A correlation analysis was performed on the one ROI in which we found a significant effect of age, in order to better characterize this significant association.

A subset of the ASD specimens (*n* = 8) were provided to us with the scores of the Autism Diagnostic Interview-Revised (ADI-R). This standardized questionnaire contains three content sections: Section A-Qualitative Abnormalities in Reciprocal Social Interaction, Section B (Verbal or Nonverbal)-Qualitative Abnormalities in Communication, and Section C-Restrictive, Repetitive, and Stereotyped Patterns of Behaviors. Higher scores on each section indicate greater impairment. For the ROIs in which we found a main effect of ASD, we performed correlations between OXTR binding density and the scores on each of the three sections of the ADI-R in order to investigate possible associations between neural measures and symptom severity. Because none of these correlations were significantly non-zero, these results are provided in the Supplementary Materials as Supplementary Figures 1 (NBM) and 2 (VP).

## Results

### Regions of specific OXTR binding

**Fig. 3:**
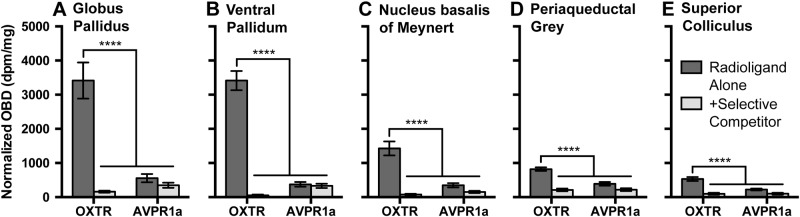
OXTR and AVPR1a radioligand binding in five regions of the human brain using competitive -binding receptor autoradiography. Optical binding density (OBD) across all regions of interest and all specimens for the OXTR radioligand alone as well as with a human OXTR-selective competitor (ALS-II-69), and for the AVPR1a radioligand alone as well as with a human AVPR1a-selective competitor (SR49059). Our approach reveals areas of putative OXTR expression in the external segment of the globus pallidus (GP), ventral pallidum (VP), nucleus basalis of Meynert (NBM), periaqueductal gray (PAG), and superior colliculus (SC) of the human brain. Data are expressed as mean ± SEM. ****p < 0.0001

In each of the five ROIs (Fig. 3), across all specimens, we found significant main effects of radioligand (GP: *F*_1,44_ = 23.58, *p* < 0.0001; VP: *F*_1,111_ = 84.64, *p* < 0.0001; NBM: *F*_1,136_ = 21.42, *p* < 0.0001; PAG: *F*_1,144_ = 19.99, *p* < 0.0001; SC: *F*_1,136_ = 16.70, *p* < 0.0001) and of competitor (GP: *F*_1,44_ = 39.64, *p* < 0.0001; VP: *F*_1,111_ = 128.4, *p* < 0.0001; NBM: *F*_1,136_ = 50.57, *p* < 0.0001; PAG: *F*_1,144_ = 68.04, *p* < 0.0001; SC: *F*_1,136_ = 55.68, *p* < 0.0001), as well as interaction effects (GP: *F*_1,44_ = 30.66, *p* < 0.0001; VP: *F*_1,111_ = 122.2, *p* < 0.0001; NBM: *F*_1,136_ = 28.21, *p* < 0.0001; PAG: *F*_1,144_ = 68.04, *p* < 0.0001; SC: *F*_1,136_ = 17.12, *p* < 0.0001). Post hoc comparisons between the OXTR radioligand-alone and AVPR1a radioligand-alone conditions revealed significantly higher OXTR radioligand binding in all five regions (GP: *t* = 7.349, *p* < 0.0001; VP: *t* = 14.39, *p* < 0.0001; NBM: *t* = 7.028, *p* < 0.0001; PAG: *t* = 6.450, *p* < 0.0001; SC: *t* = 5.816, *p* < 0.0001). Across all specimens and all five regions, after correcting for multiple comparisons, the levels of AVPR1a radioligand binding were not significantly different from the levels that resulted in the presence of either selective competitor; therefore, we cannot conclude that there is any appreciable amount of AVPR1a expression in these five areas of the human brain.

### Effects of ASD diagnosis and age on OXTR binding

**Fig. 4:**
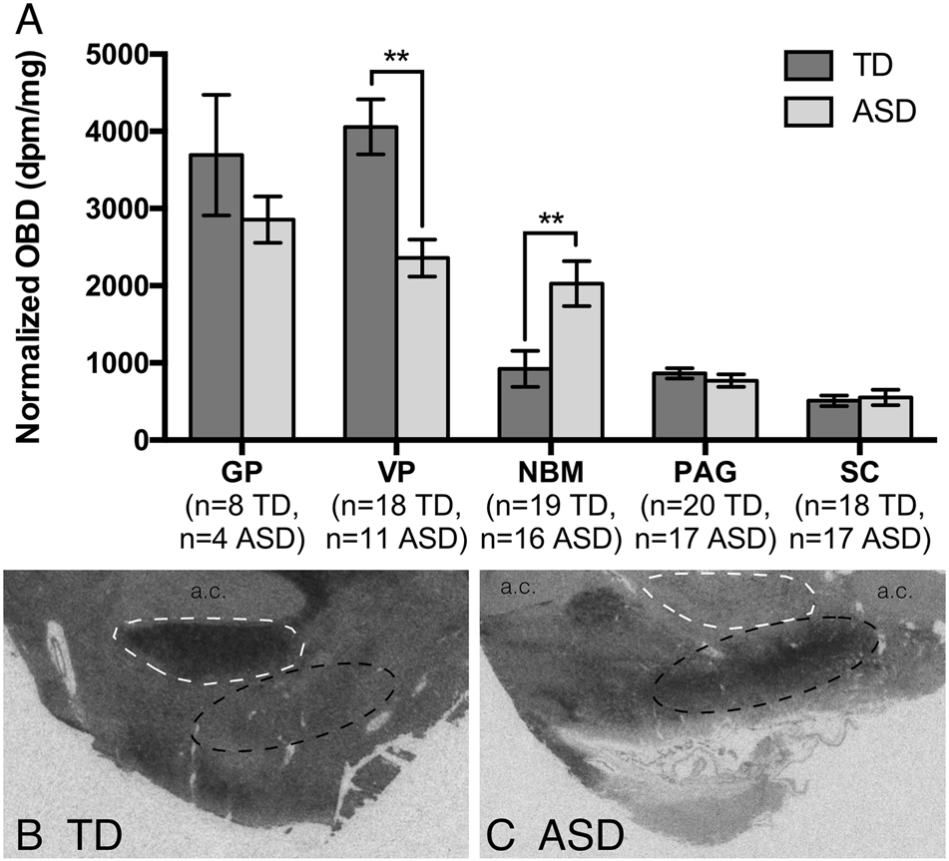
Effect of autism spectrum disorder on OXTR binding in five regions of the human brain. **a** Comparisons between OXTR binding density in typically developing (TD) and autism spectrum disorder (ASD) specimens show significant differences in the ventral pallidum (VP) and nucleus basalis of Meynert (NBM), but not in the external segment of the globus pallidus (GP) periaqueductal gray (PAG), or superior colliculus (SC). Data are expressed as mean ± SEM. **p < 0.01. **b** Representative binding in the VP (white outline) and NBM (black outline) from a TD specimen. **c** Representative binding in the VP (white outline) and NBM (black outline) from an ASD specimen

There was a significant effect of ASD diagnosis on OXTR density in two of the five ROIs (Fig. 4a): the NBM (*F*_1,33_ = 10.71, *p* < 0.01) and VP (*F*_2,26_ = 7.92, *p* < 0.01). In the NBM, ASD specimens had significantly higher OXTR binding compared to TD specimens (black outlined region in Fig. 4b, c). In the VP (white outlined region in Fig. 4b, c), ASD specimens had significantly lower OXTR binding compared to TD specimens.

**Fig. 5:**
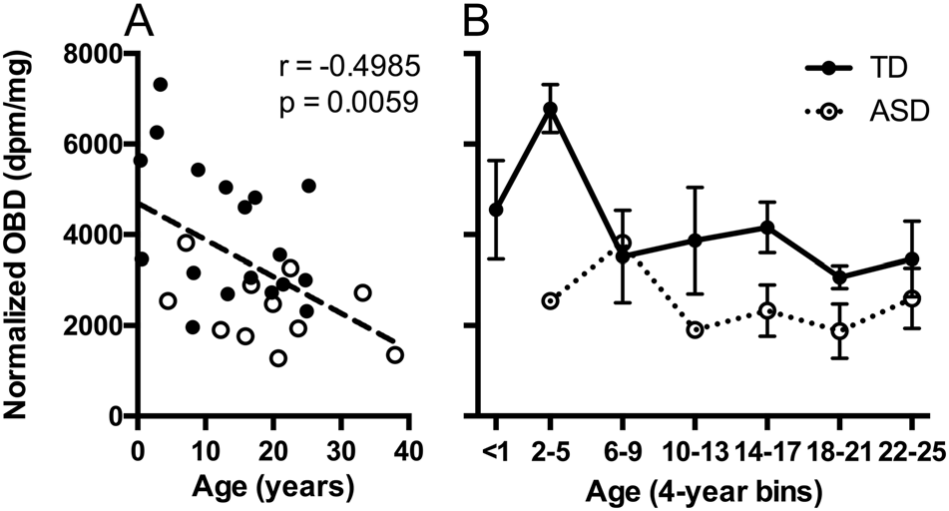
Effect of age on OXTR binding in the ventral pallidum (VP). **a** Significant negative association between age and OXTR binding across all samples. **b** Binning the data into 4-year bins suggests a potential critical period in early childhood in typically developing (TD; closed circles, solid line) specimens in which OXTR is maximally expressed in the VP—a peak that is absent, or possibly dampened and shifted later in childhood, in the specimens from individuals with autism spectrum disorder (ASD; open circles, dotted line). Data are expressed as mean ± SEM

Also in the VP, there was a significant effect of age (*F*_2,26_ = 5.38, *p* < 0.05), and further analysis of this effect found a significant negative correlation between age and OXTR density in this region (*r* = −0.4985, *p* < 0.01; Fig. 5a). This association in the VP was driven entirely by the neurotypical controls (*r* = −0.6299; *p* < 0.01) and not by the ASD specimens (*r* = −0.0902; *p* = 0.73). Although there was not a significant age × diagnosis interaction, we did find a peak in OXTR density in the VP in TD specimens around ages 2–5 yeara which was absent, or perhaps dampened and shifted toward later childhood, in ASD specimens (Fig. 5b).

In the GP, SC, and PAG, we found no significant effects of diagnosis, age, or any of the other factors and no interaction effects.

### Negative results worth noting

There was no significant effect of PMI on OXTR density in any ROI, which supports and extends the results from our previous study in postmortem human brainstem tissue^34^ and suggests that the tertiary structure and pharmacological features of OXTR remain intact across brain regions for extended periods of time after death. Despite the male bias in ASD diagnoses^53^ and a precedent from the rodent literature for sex differences in OXTR binding^54^, there were no significant sex differences in OXTR density and no significant sex × diagnosis interactions in any of the ROIs.

## Discussion

To our knowledge, this is the first report that OXTR binding is altered in specific subregions of the ASD brain. Our areas of interest were informed by recent, comparative research in primate brain tissue, because the previously published neural maps of OXTR in the human brain^35,36^ have recently been called into question due to potentially inaccurate methodological approaches. We focused on the two areas that express OXTR across all primates examined to date^39^: the NBM in the basal forebrain and the SC in the midbrain. We also opportunistically analyzed a few additional regions that are adjacent to those two ROIs, including the VP and GP in the basal forebrain and neighboring PAG in the midbrain. Interestingly, despite the established pharmacological cross-talk that the OXTR radioligand ^125^I-OVTA and the AVPR1a radioligand ^125^I-LVA exhibit in nonhuman primate brain tissue, these radioligands seem to produce selective labeling in these five regions of the human brain. We found that all five areas contained putative OXTR binding, which agrees with what was reported in the older study (mentioned above^35^) and supports the idea that these tools are more selective in the human brain compared to that in a nonhuman primate brain. Our methods did not detect AVPR1a binding in any ROI, which suggests a lack of AVPR1a expression in these regions of the human brain, although future studies that use secondary analyses, such as in situ hybridization, to confirm the absence of *AVPR1a* mRNA are recommended.

In our analysis of OXTR density across these five regions, we found a significant effect of ASD diagnosis in the VP and NBM. In the NBM, ASD specimens had significantly higher OXTR binding compared to TD specimens. The NBM modulates visual attention; it is possible that higher OXTR levels here may underlie the aberrant patterns of attention to social stimuli in ASD^45,46,55^. Alternatively, it may reflect regulatory plasticity in OXTR expression, such that this higher OXTR in ASD may be compensatory for lower levels of OXT peptide release, perhaps due to a chronic lack of engagement in OXT-releasing visual/social behaviors, like reciprocal social interaction or eye contact^56^. In the VP, ASD specimens had significantly lower OXTR binding compared to TD specimens. The VP is part of the mesolimbic dopamine reward pathway and is important in processing reinforcing stimuli; it is speculative, but low OXTR levels in this area may be related to a reduced experience of OXT-mediated social reward in ASD.

Of the five regions examined, we found a significant effect of age only in the VP, where OXTR binding decreases with age across all samples. Further analysis revealed a transient increase in OXTR binding in early life in TD specimens, which is absent in ASD specimens. This result may reflect a critical period in typical human development when this reward area is maximally sensitive to OXT in early childhood, between ages 2 and 5 years. This idea is interesting in the context of autism, because most children with ASD begin showing symptoms in this age range and typically receive an ASD diagnosis by age four^53^. However, due to this average age of ASD diagnosis, the number of specimens in the brain bank with a confirmed ASD diagnosis are limited, if not absent, at the youngest ages. Thus, we recognize that our conclusions here are based on a very small sample size, but we felt this pattern was notable and its implications are highly deserving of future research.

There has been a variety of research on gross neuroanatomical differences between people with ASD and TD individuals, but relatively little is known about specific measures of the OXT system in either ASD or TD brains. Two studies have previously examined the levels of *OXTR* gene transcripts in postmortem ASD brain specimens (in the prefrontal cortex^57^ and temporal cortex^58^). However, these studies focused primarily on the mechanisms that regulate *OXTR* gene expression (microRNAs^57^ and epigenetics^58^) and did not directly measure binding to mature, cell-surface OXTR protein, as receptor autoradiography does, which was used in this study. In addition, both studies examined only a single cortical region of interest, rather than multiple neighboring and distant areas as we did in the current study. Finally, unlike the current study, which used histological sections to preserve neuroanatomy, both of these prior studies worked with tissue homogenates. Despite these differences and limitations, the results of these studies are interesting in light of the results of the current study. Gregory et al.^58^ reported decreased *OXTR* mRNA in the superior temporal gyrus of ASD specimens, but Mor et al.^57^ reported increased *OXTR* mRNA in the prefrontal cortex of ASD specimens. We also found opposing results across regions, with significantly higher OXTR binding in the NBM, but significantly lower OXTR binding in the VP. These results point to a dysregulated OXTR system throughout cortical and subcortical areas of the ASD brain and highlight the importance of future research on the regulatory mechanisms that are responsible for these complex effects.

Although these studies and others^59–61^ indicate that *OXTR* mRNA can be measured in some portions of the human cortex, autoradiographic studies of OXTR binding have not generated complementary evidence of functional, cell-surface OXTR in these regions of the cortex in humans^35^ or in nonhuman primates^39^. This anatomical contradiction is also at odds with the numerous in vivo fMRI studies in humans that report changes in cortical BOLD signal after IN-OXT treatment (see the sixty-six publications using fMRI and IN-OXT that were included in this meta-analysis^62^). However, these seemingly opposing findings can be reconciled in light of our evidence for OXTR expression in the NBM of humans (current study and ref. ^35^) and nonhuman primates^39^. The NBM provides cholinergic input to the entire cortex^63^ as well as the amygdala and other regions of the social brain (see Fig. 5 in the recent review by Putnam et al.^64^ for an anatomical model of NBM connectivity to social neural networks). Based on this connectivity of the NBM, we and others have postulated that OXT acting at OXTR in the NBM could modulate the activity of an extensive network of brain areas (which may themselves lack OXTR) and influence social behavioral processes like social visual attention, gaze direction, and social decision making^38,39,64,65^. Indeed, emerging evidence from rhesus macaques indicates that OXT injected site specifically into the rhesus macaque NBM affects social attention, as well as neural activity in the amygdala^66^. This work provides anatomical support for other findings in macaques, which indicate that IN-OXT treatment increases gaze following^67^, increases socially oriented gaze^68^, and alters social attention in a wide variety of contexts^69–74^. Therefore, while OXT is known to act directly at specific neural OXTR populations to modulate rodent behavior, including (but not limited to) social recognition in mice^75^ and pair bonding in voles^76^, these behavioral and anatomical studies in nonhuman primates seem to indicate a more nuanced picture of central OXT action, in which OXT may be acting as “a modulator of modulators” via the cholinergic system to influence neural activity and behavior.

This model of OXT action in the primate brain is also supported by evidence from human studies and provides a theoretical framework for the neural basis of the social symptoms of ASD. IN-OXT has also been shown in an increasing number of studies to change social visual attention in non-clinical populations of humans^77–81^. It is also known that IN-OXT treatment can ameliorate^82^ the lack of attentional preference for faces^46^ that is characteristic of patients with ASD. In this context, perhaps exogenous OXT is acting at upregulated OXTR in a sensitized NBM in order to augment social function in patients with ASD. There is also neuroanatomical evidence from postmortem ASD specimens to support the theoretical framework of a dysregulated OXT-cholinergic circuit; while the basal forebrain markers of acetylcholine synthesis and breakdown were not significantly different between TD and ASD specimens, which suggests normal cholinergic innervation of the cortex in ASD, the densities of nicotinic and muscarinic acetylcholine receptors were substantially reduced in the frontal and parietal cortex of ASD specimens^83^. Taken together, it seems plausible that the social attentional symptoms of ASD could result from an altered basal forebrain circuit that involves OXT and downstream targets of the cholinergic system, especially in the cortex. While this framework does not specifically address our findings in the VP, we feel that future studies are needed to connect these systems and to provide cellular, histological evidence of the colocalization of OXT/OXTR with the cholinergic system and other neurotransmitters systems of the human brain, such as the dopaminergic circuits of the mesolimbic reward pathway.

To conclude, our results have important clinical implications and point to several directions for future research. First, we can now quantify the functional, cell-surface OXTRs in distinct regions of the ASD brain. Given the association between *OXTR* polymorphisms and ASD phenotypes, we plan to incorporate *OXTR*-related genetic measures in these specimens to potentially link genetic variation to these neural measures. If successful, these studies would provide the first gene–brain–behavior link in humans—a phenomenon that has recently been established in animals^84^. Considering the use of IN-OXT as a therapy for patients with ASD, it is important to consider that this treatment may be acting upon an already dysregulated receptor system. If these OXTR receptor densities are immutable features of the ASD brain, IN-OXT may not be as effective as once hoped. However, if these altered OXTR densities in the ASD brain are instead reflective of plasticity in the OXT system, then IN-OXT treatment may contribute to an already sensitive OXTR neural system. It may also be possible to rescue these receptor differences with behavioral interventions that are known to activate endogenous OXT release. Our results highlight the importance of continued research in this area to tackle the many remaining questions about the involvement of the OXT system in the biology of ASD.

## Electronic supplementary material

Supplemental Material
