# Supplementary material for: Effect of age and autism spectrum disorder on oxytocin receptor density in the human basal forebrain and midbrain
Source: Transl Psychiatry. 2018 Dec 4;8:257. doi: 10.1038/s41398-018-0315-3 (PMC6279786; doi:10.1038/s41398-018-0315-3)

Supplementary information for Freeman et al. Effect of age and autism spectrum disorder on oxytocin receptor density in the human basal forebrain and midbrain. TD, typically developing. ASD, autism spectrum disorder

Supplementary Table 1. Causes of death, summarized into nine main categories.

| **Cause of Death** | **# TD Specimens** | **#ASD specimens** |
| --- | --- | --- |
| Unknown/not reported | 1 | 1 |
| Drowning | 3 | 4 |
| Seizure | 0 | 1 |
| Cardiovascular | 8 | 3 |
| Asphyxia | 4 | 1 |
| Suicide by hanging | 3 | 1 |
| Physical trauma | 4 | 3 |
| Respiratory/infection | 2 | 3 |
| Cancer | 0 | 2 |

Supplementary Table 2. Sex information for the TD and ASD specimens. ASD is a on average a 4.5:1 male biased condition^3^, and our specimens reflect that, although with a slightly lower male to female ratio.

| **Sex** | **# TD Specimens** | **#ASD specimens** |
| --- | --- | --- |
| Female | 12 | 5 |
| Male | 10 | 17 |
| Male to Female Ratio | 0.8 | 3.4 |

Supplementary Table 3. Age information for the TD and ASD specimens. Although the majority of the typically TD and ASD ages overlap, the TD sample set includes five specimens that are younger than the youngest ASD specimen, and the ASD sample set includes five specimens that are older than the oldest TD specimen.

| **Age** | **TD** | **ASD** |
| --- | --- | --- |
| Age range (Years) | 0.27 - 25.27 | 4.45 - 67.33 |
| Mean age (Years+/-SD) | 13.01 +/- 8.34 | 19.89 +/- 15.34 |
| Median Age (Years) | 13.87 | 16.31 |

Supplementary Table 4. Race information for the TD and ASD specimens.

| **Race** | **# TD Specimens** | **#ASD specimens** |
| --- | --- | --- |
| African American | 10 | 9 |
| Caucasian | 9 | 12 |
| Hispanic | 2 | 1 |
| Not provided | 1 | 0 |

Supplementary Figure 1. Association between OXTR density in the nucleus basalis of Meynert (NBM) and ADI-R scores in ASD specimens (n=8).


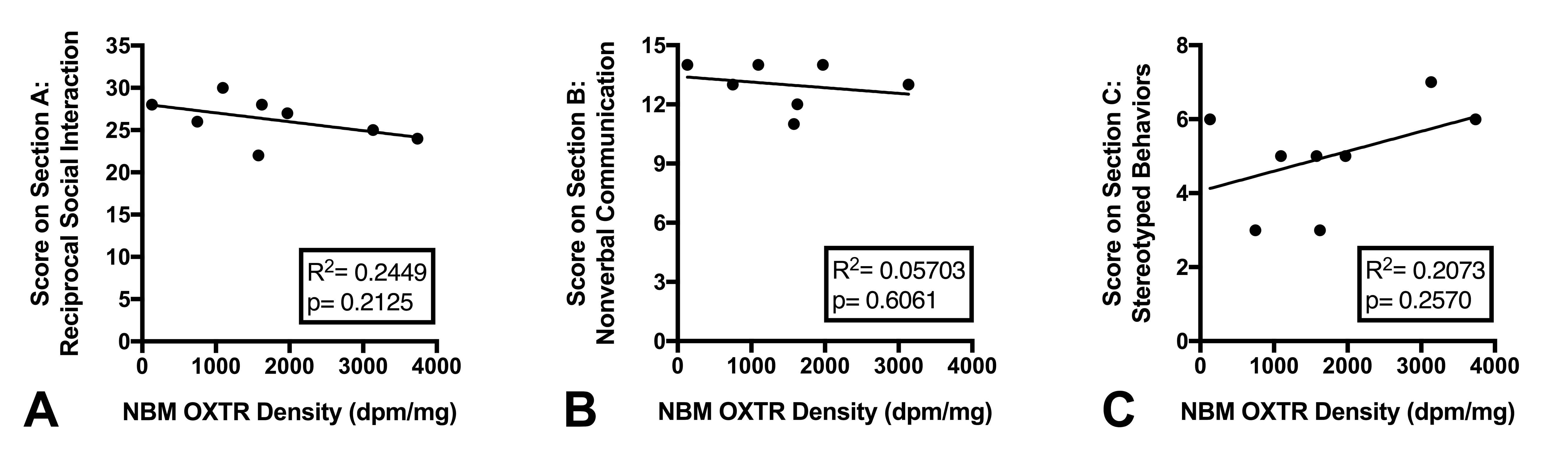


Supplementary Figure 2. Association between OXTR density in the ventral pallidum (VP) and ADI-R scores in ASD specimens (n=8).


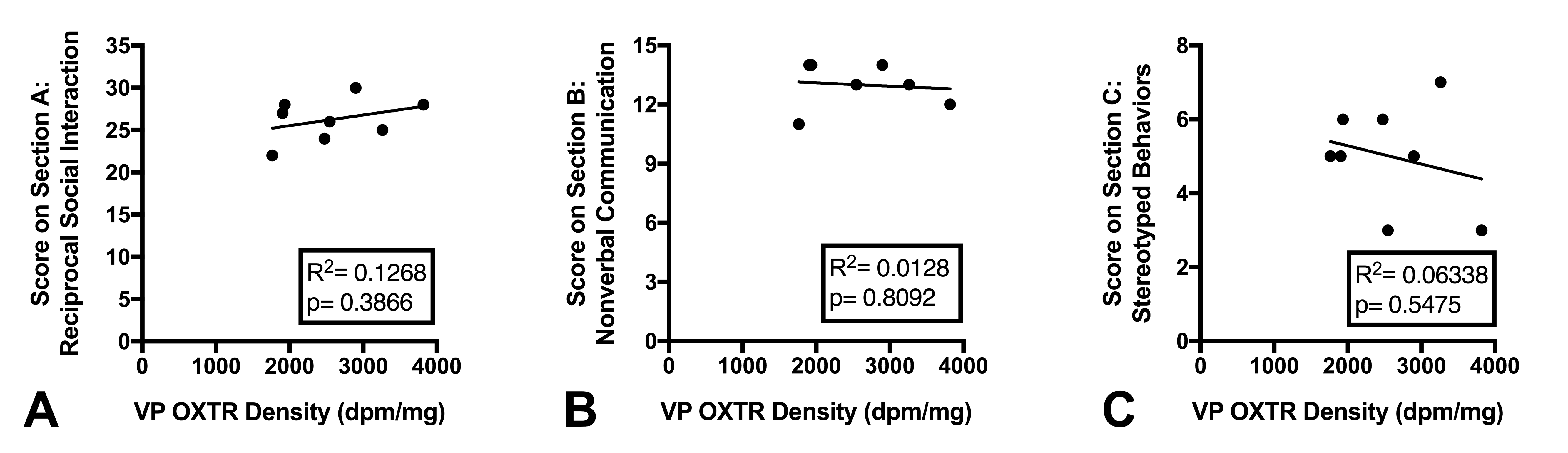

Supplement: Supplementary file 1 — Supplemental Material [file 41398_2018_315_MOESM1_ESM.docx]
